# The CO-produced Psychosocial INtervention delivered by GPs to young people after self-harm (COPING): protocol for a feasibility study

**DOI:** 10.3310/nihropenres.13576.1

**Published:** 2024-05-08

**Authors:** Faraz Mughal, Carolyn A. Chew-Graham, Benjamin Saunders, Sarah A. Lawton, Sarah Lewis, Jo Smith, Gillian Lancaster, Ellen Townsend, Christopher J. Armitage, Peter Bower, Nav Kapur, David Kessler, Alba X. Realpe, Nicola Wiles, Dennis Ougrin, Martyn Lewis

**Affiliations:** 1 School of Medicine, Keele University, Keele, England, UK; 2 School of Psychology, University of Nottingham, Nottingham, England, UK; 3 Manchester Centre for Health Psychology, School of Health Sciences, The University of Manchester, Manchester, England, UK; 4 Manchester University NHS Foundation Trust, Manchester Academic Health Sciences Centre, The University of Manchester, Manchester, England, UK; 5 NIHR Greater Manchester Patient Safety Research Collaboration, The University of Manchester, Manchester, England, UK; 6 Centre for Primary Care and Health Services Research, The University of Manchester, Manchester, England, UK; 7 Centre for Mental Health and Safety, Manchester Academic Health Sciences Centre, The University of Manchester, Manchester, England, UK; 8 Mersey Care NHS Foundation Trust, Liverpool, England, UK; 9 Centre for Academic Primary Care, Population Health Sciences, Bristol Medical School, University of Bristol, Bristol, England, UK; 10 Population Health Sciences, University of Bristol, Bristol, England, UK; 11 NIHR Bristol Biomedical Research Centre, University Hospitals Bristol and Weston NHS Foundation Trust, University of Bristol, Bristol, England, UK; 12 Centre for Academic Mental Health, Population Health Sciences, Bristol Medical School, University of Bristol, Bristol, England, UK; 13 Youth Resilience Unit, Centre for Psychiatry and Mental Health, Wolfson Institute of Population Health, WHO Collaborating Centre for Mental Health Services Development, Queen Mary University of London, London, England, UK

**Keywords:** Protocol, self-harm, young people, general practice, general practitioner, feasibility, mixed methods

## Abstract

**Background:**

Self-harm in young people is a growing concern and reducing rates a global priority. General practitioners (GPs) can intervene early after self-harm but there are no effective treatments presently available. We developed the GP-led COPING intervention, in partnership with young people with lived experience and GPs, to be delivered to young people 16–25 years across two consultations. This study aims to examine the feasibility and acceptability of conducting a fully powered effectiveness trial of the COPING intervention in NHS general practice.

**Methods:**

This will be a mixed-methods external non-randomised before-after single arm feasibility study in NHS general practices in the West Midlands, England. Patients aged 16–25 years who have self-harmed in the last 12 months will be eligible to receive COPING. Feasibility outcomes will be recruitment rates, intervention delivery, retention rates, and completion of follow-up outcome measures. All participants will receive COPING with a target sample of 31 with final follow-up data collection at six months from baseline. Clinical data such as self-harm repetition will be collected. A nested qualitative study and national survey of GPs will explore COPING acceptability, deliverability, implementation, and likelihood of contamination.

**Discussion:**

Brief GP-led interventions for young people after self-harm are needed and address national guideline and policy recommendations. This study of the COPING intervention will assess whether a main trial is feasible.

**Registration:**

ISRCTN (ISRCTN16572400; 28.11.2023).

## Plain English Summary

Self-harm, usually by medicine overdose or cutting, affects young people, their families and friends, and society. It costs the NHS around £128 million a year. Young people who self-harm experience further self-harm, anxiety and depression. The leading cause of death in young people is suicide and over half of young people who die by suicide have previously self-harmed.

Within the NHS, the GP is the most frequently contacted health professional after self-harm for young people. The GP consultation provides a crucial opportunity for early intervention to reduce further self-harm. There are at present no effective interventions for GPs to use with young people after self-harm. We thus developed with GPs and patients a new GP-led brief psychosocial treatment for young people 16–25 years after self-harm (called COPING) to be delivered across two appointments. We now aim to test, in a feasibility study, the COPING intervention in NHS general practice to determine whether a future large scale clinical trial of COPING is feasible.

We will recruit study sites from practices in the local clinical research network West Midlands and train GPs at sites. We will identify potential participants through three recruitment strategies and all participants will receive the COPING intervention. We will collect participant follow-up data at two, four, and six months from recruitment, and data will include information about mood, self-harm thoughts, and episodes of repeat self-harm. GPs and participants will be invited for an interview to gain their experiences of COPING. An online survey of GPs in England will gauge interest about engagement in a future trial.

This study will ascertain whether COPING can be evaluated in a large scale clinical trial in NHS general practice. If feasible, acceptable, and deliverable, there is the potential for COPING to substantially benefit patients, GPs, and the NHS.

## Introduction

### Background and rationale

Self-harm, defined as self-injury or self-poisoning irrespective of the purpose, impacts on patients, families, and health and care services ^1, 2^. In young people, there is a lifetime self-harm prevalence of 26% in England, rising to 47% in those with mental illness ^3, 4^. The annual cost of hospital treated self-harm in the National Health Service (NHS) is estimated to be £128 million ^5^.

Self-harm is closely associated with death by suicide, and in young people self-harm is a major risk for premature death with around 40 years of life lost to ‘external-cause’ death ^6, 7^. General practitioners (GPs) are often the first point of healthcare access for young people after self-harm and young people who present to general practice after self-harm are 17 times more likely to die by suicide ^7^. GPs are well placed and have some skills to manage self-harm early in primary care: practice nurses have reported not feeling competent, and NHS Talking Therapies do not offer therapy for self-harm ^8^. However, at present, there are no effective treatments that GPs can offer young people after self-harm ^9^.

Reducing self-harm in young people is an international priority ^2^. There is evidence psychosocial treatments, such as cognitive behaviour therapy-based psychotherapy and dialectical behaviour therapy, can reduce repeat self-harm episodes in young people but these were delivered in non-general practice settings ^10, 11^. To address this intervention evidence gap, we developed in partnership with young people with lived experience of self-harm and GPs, a brief GP-led psychosocial intervention (COPING) to help young people aged 16–25 years, across two GP consultations, avoid future self-harm (COPING-ID study, IRAS 294180).

Through early intervention in general practice there is potential to prevent long-term self-harm in young people, reduce NHS self-harm treatment costs, and future mental health and specialist (emergency, paediatric, medical) care costs. If the COPING intervention is feasible and acceptable, a future main randomised controlled trial (RCT) has the potential to bring substantial benefit to patients and the NHS. This feasibility study aims to examine aspects of the feasibility of future pragmatic RCT to assess the effectiveness of COPING in the NHS and inform its design.

### Objectives

To examine aspects of the feasibility of a fully powered RCT of the COPING intervention across the areas of recruitment, delivery, and retention and follow-up. Specific objectives are:

i) Estimate participant consent and willingness of enrolled participants and GPs to be randomised in a future RCT
ii) Assess GP fidelity to COPING delivery and participant adherence to COPING
iii) Examine completeness of participant follow-up data collection
iv) Explore the acceptability of COPING for both participants and GPs, and deliverability of COPING for GPs

## Methods

### Study design

This study is a mixed-methods external non-randomised before-after single arm feasibility study.

This study is registered on ISRCTN registry, 28.11.2023: ISRCTN16572400; https://www.isrctn.com/ISRCTN16572400.

### Study setting

NHS general practices in the West Midlands, England.

### Eligibility criteria

Patients will be eligible to participate if they are aged 16–25 years and have self-harmed (according to the definition of the National Institute for Health and Care Excellence) in the last 12 months ^1^. Exclusion criteria include acute risk of suicide or harm to others necessitating urgent referral/admission; moderate to severe learning disabilities; current psychotic episode or organic mental illness; receiving current psychological therapy; patient inappropriate for COPING at clinician discretion; and lacking capacity for informed consent.

At general practice study sites, GPs will be eligible to deliver COPING if they complete the site COPING training.

### Intervention

All participants will receive the COPING intervention delivered by GPs to young people aged 16–25 years after self-harm across two GP appointments. COPING’s development was guided by the new Medical Research Council’s (MRC) framework for developing and evaluating complex interventions ^12^. COPING was developed through a ‘combination’ development approach (O’Cathain’s taxonomy - integrating both theory and evidence-based and partnership approaches) ^13^. It was informed by the Behaviour Change Wheel, and included coproduction and prototyping (Hawkin’s framework) in its design and refinement ^14, 15^.

COPING comprises eight behaviour change techniques (BCTs) aimed at influencing the selected target behaviours for the young person and the GP. The target behaviours are: i) the young person using new skills to avoid self-harm and ii) the GP using COPING in usual NHS practice. The BCTs are problem solving, self-monitoring of behaviour, goal setting behaviour, review behaviour goal, social support (practical), instruction on how to perform the behaviour, demonstration of the behaviour, and rewarding completion. In the first COPING appointment the GP and participant agree on new skills aligned to the function of the self-harm to use when urges of self-harm occur, and a diary is provided. In the second appointment the GP reviews progress with the participant. The first appointment will be in-person and the format and time (from two to four weeks) of the second COPING appointment will be decided between the GP and participant.

The COPING training will be delivered by the Chief Investigator (CI) to site GPs in person and last up to 60 minutes. The training will include presenting the study background (including COPING-ID), rationale and aim, providing the COPING intervention materials, offering to show an example COPING delivery video, and highlighting study procedures and safety reporting.

### Outcomes

The primary outcomes are:

- Consent to study of eligible patients
- Young people participants willing to be randomised in a main RCT
- Fidelity to COPING
- Participant adherence to COPING
- Participant follow-up rate of outcome measures
- Acceptability of COPING to participants and GPs
- Deliverability of COPING

The secondary outcomes are:

- Response rates of invited practices to participate
- Identification of patients at sites
- Proportion of identified patients eligible for study inclusion
- Participant recruitment uptake through recruitment strategies
- Willingness of GPs to be randomised in a main RCT
- Assessment of outcome measure to use as the primary outcome in a main RCT
- Proportion of participants with repetition of self-harm
- Participant attrition, follow-up, and withdrawal rates

This feasibility study is not designed to evaluate health outcome measures formally. We will collect clinical effectiveness data that may support the justification for a future main trial. These data may also be used to decide which outcomes could and should be collected in a future RCT, and to estimate parameters required to calculate the sample size calculation for a fully powered RCT.

The progression decision-making process will include consideration of the feasibility study progression outcomes (aligned to the primary outcomes: see Table 1) with the study steering committee (SSC), patient and public involvement (PPI) group, and research team to consider if moving to a fully powered RCT is feasible.

**Table 1.** Progression criteria for a future main trial.

Criteria	Go – proceed to RCT	Amend – proceed with minor changes	Stop – do not proceed unless major changes are possible
Consent to study in eligible patients	>30%	15-30%	<15%
Young people participants willing to be randomised in a future RCT	>85%	60-85%	<60%
Fidelity to COPING treatment checklist	>67%	40-67%	<40%
Participant adherence to COPING	>67%	40-67%	<40%
Participant follow-up rate of outcome measures	>80%	50-80%	<50%
Acceptability of COPING to GPs and patients	COPING judged very acceptable by GPs and patients by qualitative data	COPING judged acceptable by GPs and patients by qualitative data	COPING judged somewhat acceptable by GPs and patients by qualitative data
Delivery of COPING by GPs	Delivery of COPING judged strongly feasible by qualitative data	Delivery of COPING judged feasible by qualitative data	Delivery of COPING judged possibly feasible by qualitative data

### Participant timeline

The schedule of study events is listed in Table 2.

**Table 2.** Schedule of study events.

Schedule of events		Enrolment	Baseline data collection	COPING delivery		Follow-up		
		0 weeks	0 weeks	T1	T2	8 weeks	4 months	6 months
Enrolment								
	Eligibility screen	✓						
	Informed consent	✓						
Participant descriptors								
Demographics	DOB, sex, gender		✓					
Language	Main language		✓					
Ethnicity	Ethnic origin		✓					
Education status	Type		✓					
Employment status			✓					
Internet use and access	Frequency and method		✓					
Medications								
Prescribed medications	Medication name, dosage, and frequency		✓					
Clinical outcomes								
Past self-harm	Method, timing of last self-harm, and frequency of episodes		✓					
Mood and suicidal or self-harm thoughts	PHQ-9		*✓*			✓	✓	✓
Attempted suicide	SBQ-R		✓			✓	✓	✓
COPING delivery								
Duration	Length of consultations (minutes)			✓	✓			
Deciding skills				✓	✓			
Goal setting				✓	✓			
Provision of COPING diary				✓				
Attendance at 2 ^nd^ COPING consult					✓			
Review of diary					✓			
Interviews						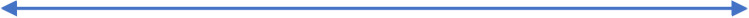		
Adverse events								
Recording of adverse events	Classifying and logging adverse events			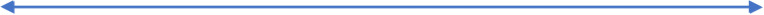				

T _1_ – first COPING consult, T _2_ – second COPING consult

### Sample size

Non-randomised COPING delivery:

We will aim to recruit approximately six general practice sites and around 3 GPs at each site. The incidence rate of recorded self-harm in young people in general practice is around 60 per 10,000 person-years ^16^. This equates to 60 per 1,000 for the 16–25 age group over one year. If half are eligible for the study (30 per 1,000) and two-thirds of those are identified by our recruiting methods (20 per 1,000) then in a typical GP practice (assumed 6,000 registered patients of which 900 (15%) are in the 16–25 age group) about 18 eligible young people will contact their GP practice per year after self-harm (1.5 eligible young people per practice per month). If six practices are recruited to take part, nine young people would be expected to be eligible per month, and around 104 (the target eligible population size) over about 12 months. From this number (n=104) we would expect to recruit 31 participants (30%) (fulfilling the Go signal for progression for the recruitment criterion – see Table 1) into the study.

The sample size of n=31 participants is required to test against the feasibility progression criteria (specifically the cut-off for a STOP signal) with at least 90% power, using the normal approximation method for binary outcomes using a 1-sample 1-sided test with 5% alpha (type I error) using the approach of Lewis *et al.* ^17^ In line with the recommendation of the CONSORT-extension for randomised pilot and feasibility studies this provides necessary justification for the sample size requirement for the evaluation of the progression criteria for this feasibility study ^18^.

GP survey:

A sample of 100 GPs across England will be surveyed to elicit GP views about a future main RCT of COPING. Achieving 100 GP responses from a convenience sample has recently been shown to be achievable ^19^. If we expect 50% to respond then n=50 would be sufficient to provide a margin of error no greater than 0.1 standard error (SE) around an estimate for a proportion based on a binary response item “e.g., would you be willing to participate in a future RCT of COPING (yes/no)”. Therefore, if 25/50 respond ‘yes’, it will generate an estimate of the proportion who answer positively of 0.5 (50%) with margin of error <= +/-0.1 relating to a z-value of +/-1.282 allowing a 90% 1-sided confidence interval (CI) and 80% 2-sided CI (providing sufficiently precise confidence interval estimation) around the observed estimate.

Nested qualitative study:

It is anticipated that a sample of around 16–20 participants (8–10 young people participants and 8–10 GPs) is likely enough to identify problems in COPING delivery and achieve data saturation, where data no longer offers new insights ^20^.

### Recruitment

General practices in the National Institute for Health and Care Research Local Clinical Research Network (CRN) West Midlands will be invited to participate in the study by email. Practices who agree to participate will receive a local information pack, a site initiation visit, COPING training, and an investigator site file. Recruited practices therefore become study sites. Each site will have a designated principal investigator (PI), and each site PI will identify GPs who want to deliver COPING to participants. GPs who use COPING with participants become study co-investigators

Patient recruitment to self-harm clinical trials in NHS general practice is untested and thus we will test three participant recruitment strategies which have been informed by the study’s PPI group:

1) General practice electronic healthcare records: practice database searches will be developed for sites to use to identify potentially eligible patients.
2) Study advertisement: recruitment posters will be displayed in site waiting rooms, linked community pharmacies, and nearby educational institutions to share the study opportunity.
3) Routine clinical practice: members of the clinical team can signpost potentially eligible patients to the study opportunity during routine consultations.

In strategy one, potential eligible patients will be identified at research sites through searching practice-based electronic healthcare registers using a standardised strategy. A practice clinician will screen the identified list of potentially eligible patients and exclude those ineligible. The remaining eligible patients, with a prior telephone check where appropriate, will be sent a text message and postal invitation from the practice signposting them to the study webpage, where the participant information sheet (PIS) and e-consent form can be found. A reminder will be sent after two weeks if no response. A simplified two-page PIS summarising what will be required from participants enrolled in the study will be made available on the study website.

Other potentially eligible patients may also be approached opportunistically by a member of the clinical care team in routine practice (strategy three). These patients and those who see the study advertised in the community around participating sites (strategy two) will be able to scan the study QR code to complete an online expression of interest. If deemed eligible for the study, they will be invited to consent into the study.

A convenience sample of GPs around England will be sought to complete an online survey. GP participants will be recruited through the professional networks and social media. GPs will have the option to be entered into a random draw for two £25 e-vouchers on completion of the survey. Young people participants who have given consent for interviews will be purposively sampled across practice location and deprivation and GPs who have delivered COPING at sites will be invited for interview and asked to e-consent. Both will be reimbursed for their time on completion of interviews.

### Patient and Public Involvement

The design of this study was informed by PPI through a self-harm in young people PPI advisory group led by the CI at Keele University, and by consulting the McPin Foundation’s Young People’s Advisory Group and the Kings College London/NIHR Maudsley Biomedical Research Centre Young Person’s Mental Health Advisory Group. This feasibility study will be supported by the COPING study PPI advisory group consisting of young people who self-harm and carers of young people who self-harm. This group supported the COPING-ID study.

The first study meeting occurred on 9 May 2023 where all members of the advisory group attended, and the group helped design the participant eligibility and study progression criteria. Members also gave feedback about the content and design of the participant study information sheet and participant recruitment poster. The group will meet two monthly to support the delivery and dissemination of this study.

### Data collection methods

Patients who consent to participate will be asked to complete an online baseline questionnaire which will collect data on participant age, sex, ethnicity, medication, number and time of recent self-harm episodes, the patient health questionnaire (PHQ-9), the Suicidal Behaviours Questionnaire-Revised (SBQ-R), and educational status.

Participant follow-up outcome data collection will be at eight weeks, four, and six months, from baseline using a self-report electronic questionnaire. Data collected will include individual participant repetition of self-harm via the SBQ-R, and PHQ-9 scores will be attained at these timepoints. Participants will be asked if they would be willing to be randomised in a future RCT of COPING in the eight-week follow-up questionnaire. One email or SMS text message reminder will be sent after one week if there has been no response after each questionnaire invitation. If a participant withdraws from the study, their data will be retained and used for the study. In the situation a participant withdraws from completing the COPING treatment, they will still be invited to complete the follow-up questionnaires.

GPs will record training time, length of COPING consultations, and attendance of participant at 2nd COPING consult on Case Report Forms (CRF). General practice medical records of participants will be reviewed and relevant aspects extracted and depersonalised at participant sites, and securely transferred to the study team to obtain information for example about past medical history and prescriptions.

GPs around England will be invited to complete an online survey. This survey will ask GPs about the number of self-harm presentations in young people 16–25 years they see each month; willingness to be involved in a future RCT, including being randomised; barriers and enablers to COPING implementation; and likelihood of contamination in a future individually randomised RCT.

In the nested qualitative study, semi-structured interviews with participants and GPs will explore acceptability of COPING and acceptability of COPING consults being recorded in a future trial, potential unintended harms, willingness to take part in a future trial and to be randomised, ‘what is usual care’, barriers and enablers for COPING implementation, and views on outcome selection and measurement. The interview topic guide will be sensitised by the Theoretical Framework of Acceptability and Theoretical Domains Framework and iteratively revised ^21, 22^. FM will conduct interviews face-to-face at sites or remotely. Interview participants will be able to withdraw their data up to one week after the interview. 

### Data management

Data will be collected in electronic and paper form. Data from CRFs at sites will be stored on secure Keele University electronic servers. Participant self-report data will be collected electronically via the Keele REDCap and likely facilitated by Keele Health Survey. Transfer of data between research sites and the COPING study team will be conducted via secure email transfer.

Interviews will be audio-recorded and audio files immediately transferred onto Keele’s secure network. Audio files will be securely shared with The Transcription Company, a third-party company with whom Keele University have established data protection agreements. A dedicated password restricted study database will be developed and maintained on Keele University servers, managed by the Keele University Information & Data Security department, and will be the final repository for the data collection. Each participant will be allocated a unique study number, so that only anonymised data are used for analysis. Depersonalised medical records will be linked to a participant’s unique study number and other participant data collected.

All data will be stored on Keele University storage services within the UK and protected by industry standard security tools. All confidentiality arrangements adhere to relevant data protection regulations and guidelines (Data Protection Act 2018, UK GDPR, Caldicott, General Medical Council (GMC), Medical Research Council (MRC) UK Policy), the Confidentiality NHS Code of Practice, and the CI (FM) and Data Custodian have responsibility to ensure the integrity of the data and that all confidentiality procedures are followed. 

### Statistical methods

Participant characteristics, eligibility and recruitment rates, attendance at second COPING consult, baseline and follow-up questionnaire response rates, GP survey responses, and resource use will be reported descriptively using SPSS (Statistical Package for the Social Sciences) v27 or later ^23^. Continuous variables (e.g., age) will be reported with means and standard deviations or median (IQR) as appropriate and categorical variables (e.g., sex) will be reported with frequencies and percentages.

Analysis of outcomes against the predefined feasibility study progression criteria will include estimated percentages with 95% confidence intervals (e.g., rates of consent or willingness to be randomised). Participant follow-up rate of outcome measures will be assessed as the total completions attained of the SBQ-R measure at all three timepoints as a percentage of the possible denominator (n=93). Participant adherence to the COPING intervention will be ascertained by examining the proportion of participants who attend for the 2nd COPING consult. Participant PHQ-9 and SBQ-R scores at baseline and follow-up timepoints will be compared using paired t-test (if normal distribution) or Wilcoxon paired rank test ^24^.

Fidelity of the COPING intervention will be measured retrospectively by reviewing CRF data completed by site GPs against the COPING delivery checklist. Overall fidelity will be calculated as a percentage of the participants where the number of COPING items successfully delivered is at least three out of five (from the five BCTs targeting the young person’s target behaviour), as agreed with the SSC.

We will investigate how much missing data there is in this study to inform the planning of a future RCT. This will occur at two levels: i) observe how much loss to follow-up there will be (participant attrition or withdrawal), and ii) assess completion rates, including missing data for specific data items, of follow-up questionnaires.

### Qualitative analysis

Interview data about the acceptability and deliverability of COPING, and free text responses from the survey, will be analysed by practical thematic analysis and themes mapped to both the Theoretical Domains Framework and Theoretical Framework of Acceptability to support the identification of barriers and facilitators to COPING implementation in the NHS and COPING acceptability ^21, 22, 25^. The CI (FM) will lead analysis and it will be facilitated by NVivo 12 or later ^26^.

### Integration of data

Quantitative and qualitative data will be integrated using a triangulation protocol to enhance the validity of findings and assess data complementarity, convergence, and dissonance across key findings ^27^.

### Data monitoring and auditing

The CI will manage this study day-to-day and will convene a Study Management Group (SMG) who will be members of the research team to support study delivery. The SMG will be responsible for monitoring study progress and conduct, study protocol implementation, and that the safety and wellbeing of participants is maintained where appropriate. The SMG will escalate any concerns to the SSC and sponsor as required. The CI will meet the SMG at monthly study meetings with communication facilitated by email where needed.

The SSC, which includes an independent statistician, a GP clinical trialist, and a GP researcher, have approved the finalised study protocol, agreed meeting schedules, understand reporting lines to the study sponsor and funder, and agree to the SSC terms of reference. The role of the SSC is to ensure the study is delivered ethically and safely, and to consider the study’s progress in light of new information. The SSC is independent from the sponsor and members have declared no conflicts of interest. The SSC will meet with the CI and some members of the SMG at least once a year. Through the chair of the SSC, the SSC will make recommendations to the CI, SMG, and sponsor, and if early study termination is felt appropriate, the chair will inform the study sponsor.

Authorised representatives of the study sponsor will have access to study data for monitoring and auditing purposes. The CI will conduct audits across the research study following a risk-proportionate approach.

### Harms

The CI will monitor and log all adverse events (AEs) and serious adverse events (SAEs), including their assessment of expectedness (and relatedness where needed), in accordance with the protocol which is in agreement with Keele University Health and Social Care standard operating procedure RM16 Safety Reporting for non-CTIMP research using the study’s CRFs for AEs and SAEs. At site initiation visits, site PIs will be appropriately trained and made aware of this study’s safety reporting requirements. Where a SAE is identified at sites, it must be reported to the study team within 24 hours. Due to the nature of this study, engagement in acts of self-harm from participants during the study is to be expected.

### Ethics and dissemination

This study has received ethics approval from NHS East of England - Cambridge East Research Ethics Committee on 13.11.2023 (23/EE/0238) and Health Research Authority and Health and Care Research Wales Approval on 14.11.2023 (IRAS 327529).

There is risk that participants may describe self-harm or suicidal intent when returning self-report questionnaires or while partaking in an interview. Two risk protocols have been developed. Should likely risk to participants be identified, the relevant risk protocol will be activated, and the participant's GP notified. The protocols outline when breaking confidentiality may be considered. The CI is a GP experienced in assessing risk of self-harm or suicide in patients and all participants will receive a list of free support services for use when needed.

The final full study report will be available from the CI, and findings will be prepared for submission to peer-reviewed journals. All publications, presentations, and correspondence generated will acknowledge NIHR as the funding source. Authorship will be determined in accordance with The International Committee of Medical Journal Editors criteria, and other contributors acknowledged, on manuscripts submitted for peer-reviewed publication.

The results of the study will be available to all stakeholders in ways that are easy to access at no cost. The PPI group will advise on how to best design and disseminate results to the wider public. The findings will also be published on the COPING study website. Avenues of dissemination include open-access peer-reviewed publications in academic journals, infographics for social media, presentations at clinical, public, and academic conferences, and NHS, NIHR, professional and public networks.

## Discussion

This study will determine the feasibility of conducting a fully powered effectiveness RCT of the COPING intervention in the NHS. The COPING intervention is a novel GP-led intervention that aims to address the intervention evidence gap for young people after self-harm. It was designed through coproduction with patients and GPs and its implementation in usual NHS general practice was considered from the start.

This feasibility study is designed to be pragmatic and deliverable in usual NHS general practice and has been informed by PPI. Three recruitment strategies will be tested to enhance understanding about identifying potentially eligible participants and the study’s progression criteria will determine whether a future main RCT is justified. Qualitative data will provide insights into how to optimise COPING and improve its delivery in practice. A national survey of GPs will provide data to help consider the design of an RCT. The integration of different types of data will enhance the credibility of findings.

The 2023–2028 suicide prevention in England strategy states that a priority area is for tailored support to be available to priority groups ^28^. Young people who have self-harmed are a high-risk priority group (encompassing two priority groups: young people and people who have self-harmed) and therefore if acceptable, deliverable, and feasible to be tested in a fully powered RCT, COPING has the potential to benefit patients, GPs, and the NHS, and address this national priority area.

## Ethics and consent

This study has received ethics approval from NHS East of England - Cambridge East Research Ethics Committee on 13.11.2023 (23/EE/0238) and Health Research Authority and Health and Care Research Wales Approval on 14.11.2023 (IRAS 327529). Participants will provide written informed consent via the study’s e-consent form.

## Data Availability

No data are associated with this manuscript. This manuscript has been written to include the recommended items from the SPIRIT 2013 checklist for interventional trials.
