# Supplementary material for: The CO-produced Psychosocial INtervention delivered by GPs to young people after self-harm (COPING): protocol for a feasibility study
Source: NIHR Open Res. 2024 Oct 15;4:27. Originally published 2024 May 8. [Version 2] doi: 10.3310/nihropenres.13576.2 (PMC11503009; doi:10.3310/nihropenres.13576.2)
Supplement: Peer review history

# Reviewer report: Reviewer response for version 2

**DOI:** 10.3310/nihropenres.14958.r33126

**Published:** 2024-10-24

**Authors:** Maria Livanou

**Affiliations:** 1 Department of Psychology, King's College London, London, England, UK

The authors have addressed all previous comments and the manuscript has been amended substantially.

Is the study design appropriate for the research question?

Yes

Is the rationale for, and objectives of, the study clearly described?

Yes

Are sufficient details of the methods provided to allow replication by others?

Partly

Are the datasets clearly presented in a useable and accessible format?

Partly

Reviewer Expertise:

child and adolescent mental health

I confirm that I have read this submission and believe that I have an appropriate level of expertise to confirm that it is of an acceptable scientific standard.

# Reviewer report: Reviewer response for version 2

**DOI:** 10.3310/nihropenres.14958.r33125

**Published:** 2024-10-17

**Authors:** Karen Rodham

**Affiliations:** 1 Department of Health Psychology, University of Chichester, Chichester, England, UK

Thank you for simplifying the lay summary. 

 I have no further comments to make.

Is the study design appropriate for the research question?

Yes

Is the rationale for, and objectives of, the study clearly described?

Yes

Are sufficient details of the methods provided to allow replication by others?

Yes

Are the datasets clearly presented in a useable and accessible format?

Not applicable

Reviewer Expertise:

I have experience of researching young people who self-harm. I am a qualitative researcher with a track record of exploring how we can better help people to live well in spite of complex (multiple) physical health conditions.

I confirm that I have read this submission and believe that I have an appropriate level of expertise to confirm that it is of an acceptable scientific standard.

# Reviewer report: Reviewer response for version 1

**DOI:** 10.3310/nihropenres.14737.r31764

**Published:** 2024-08-23

**Authors:** Maria Livanou

**Affiliations:** 1 Department of Psychology, King's College London, London, England, UK

I would like to thank the authors for this important work, given the alarmingly high numbers of young people who self-harm. This study protocol outlines the procedures involved in testing the feasibility of the COPING intervention, designed to be delivered by general practitioners (GPs) over two consultations to reduce self-harming in young people aged 16-25 years. COPING was co-produced with young people with lived experience and GPs. The present feasibility trial aims to examine the acceptability of conducting a fully powered trial.

**Abstract**

 Background

 What about the increasing numbers in the UK? Any specific trends that could be reported here to showcase the magnitude of the problem?

 Methods

  

- It would help if the authors were specific about the number of GP practices involved in the feasibility study.

Discussion

  

- “Brief GP-led interventions for young people after self-harm are needed and address national guideline and policy recommendations”. Do the authors mean to address?

 

- Any expected outcomes?

**Plain English Summary**

 “Young people who self-harm experience further self-harm, anxiety and depression”.

 Do the authors mean that young people who self-harm are more likely to repeat such behaviours in the future?

 The costs of self-harming would be a better fit in the second paragraph as the first one presents and describes the problem of self-harming. Here the consequences and impact on the young people and their families would be more relevant.

**Introduction**

 The authors mention that 47% of those who self-harm have a mental illness. It would help if they were specific about which ones are related to self-harming. E.g., depression, eating disorders.

 The types of self-harming should be addressed in more depth and, especially some forms not widely known such as picking skin, hair pulling, which are very prevalent in young people. Then, it is important to discuss why young people might be engaging in self-harming behaviours.

 The introduction would benefit from including specific trends in self-harming with regards to demographic characteristics (e.g., age groups, gender, ethnicity) and clinical features (e.g., depression, anxiety, eating disorders). For example, young people living in deprived areas, those with chronic mental health conditions such as anorexia nervosa, those who have lost a parent, and those in foster care might be presenting with a higher risk for self-harming. I think such social determinants are crucial when looking at self-harming in young people.

 The authors refer to GPs as having some skills to manage self-harm so I wonder about training in GPs in detecting and exploring self-harm. Do they receive adequate training to address and cope with such issues?

 Talking therapies might not be offering specific targeted interventions but self-harm is a symptom so the other risk factors in a young person’s life may be addressed.

 It would be helpful to explore more what elements of existing evidence-based interventions and approaches have informed the current intervention. What is the framework other than co-production? The co-production process could be described to show how this led to the development of COPING.

 Methods

 Eligibility criteria

 How will the authors define self-harming?

 I wonder what is the role of carers in the process e.g., parents and how they can be involved at least in the qualitative phase of the study.

 A table outlining the design and format of the intervention would be helpful and also the 8 behaviour change techniques and how these will be applied across the two consultations.

 Patient and Public Involvement

 This section could include further information about the Steering Groups and how the content of the intervention was developed.

Is the study design appropriate for the research question?

Yes

Is the rationale for, and objectives of, the study clearly described?

Yes

Are sufficient details of the methods provided to allow replication by others?

Partly

Are the datasets clearly presented in a useable and accessible format?

Partly

Reviewer Expertise:

child and adolescent mental health

I confirm that I have read this submission and believe that I have an appropriate level of expertise to confirm that it is of an acceptable scientific standard, however I have significant reservations, as outlined above.

# Response

**Published:** 2024-10-10

**Authors:** Faraz Mughal

**Affiliations:** School of Medicine, Keele University, Keele, England, UK

We thank the reviewer for their positive comment on our manuscript and work.

**What about the increasing numbers in the UK? Any specific trends that could be reported here to showcase the magnitude of the problem?**

 We have now added the following sentence to address this point:

 ‘Rates of self-harm documented in general practice has been increasing for young people in the UK in the last two decades, especially in 13–16-year-olds.’

**It would help if the authors were specific about the number of GP practices involved in the feasibility study.**

 This is a protocol manuscript and so we cannot say for certain how many practices will actually be involved (only what number we originally planned for). We mention in the methods of the manuscript that we aimed to recruit up to six practices. In the publication of the feasibility study findings the exact number of practices recruited will be stated.

 Thank you. We have reworded to ‘to address’.

**Any expected outcomes?**

 We are limited by the stipulated word count of the abstract for the journal but we do list intended primary and secondary study outcomes in the methods of the manuscript.

**Plain English Summary**

**“Young people who self-harm experience further self-harm, anxiety and depression”. Do the authors mean that young people who self-harm are more likely to repeat such behaviours in the future?**

**The costs of self-harming would be a better fit in the second paragraph as the first one presents and describes the problem of self-harming. Here the consequences and impact on the young people and their families would be more relevant.**

 We have reworded this sentence in response to this comment to the following as below:

 ‘Young people who self-harm are more likely to self-harm again and suffer from anxiety and depression’

 We have now moved the sentence about the costs of self-harm to the NHS to the second paragraph.

**Introduction**

**The authors mention that 47% of those who self-harm have a mental illness. It would help if they were specific about which ones are related to self-harming. E.g., depression, eating disorders.**

 We have reworded this now to read ‘rising to 47% in those with a mental health disorder such as anxiety or depression’. The national youth survey done in England from where this data comes from defined disorder according to ICD-10 categories.

**The types of self-harming should be addressed in more depth and, especially some forms not widely known such as picking skin, hair pulling, which are very prevalent in young people. Then, it is important to discuss why young people might be engaging in self-harming behaviours.**

 We have now added in the second paragraph of the introduction the common methods of self-harm in young people noted in the literature such as skin pinching and hair pulling, and have described the functions of self-harm in 16-25 year olds.

**The introduction would benefit from including specific trends in self-harming with regards to demographic characteristics (e.g., age groups, gender, ethnicity) and clinical features (e.g., depression, anxiety, eating disorders). For example, young people living in deprived areas, those with chronic mental health conditions such as anorexia nervosa, those who have lost a parent, and those in foster care might be presenting with a higher risk for self-harming. I think such social determinants are crucial when looking at self-harming in young people.**

 We agree with the reviewer that these social determinants are important in young people who have self-harmed. We have included one new sentence about the trends in rates of self-harm recorded in general practice in recent years which we feel add to the background of self-harm presentations in young people in general practice leading up to the rationale of this planned feasibility study. We have also added one sentence about likelihood of self-harming behaviour and socio-economic status and family structure in young people with a new reference from a 2017 Public Health England report.

**The authors refer to GPs as having some skills to manage self-harm so I wonder about training in GPs in detecting and exploring self-harm. Do they receive adequate training to address and cope with such issues?**

 Thank you for this point. All qualified GPs should be competent in identifying and managing patients after self-harm in daily consultations – this is part of their core training to become GPs in the UK. However, the confidence of GPs varies in handling self-harm in young people in practice which is highlighted by past research (we have added one new reference to strengthen this point). This was a key factor in developing the COPING intervention: to improve GP confidence in handling self-harm in young people in practice.

**Talking therapies might not be offering specific targeted interventions but self-harm is a symptom so the other risk factors in a young person’s life may be addressed.**

 We acknowledge that self-harm can be a result of underlying mental illness such as depression, anxiety, or post-traumatic stress, and so NHS talking therapy services may be beneficial, however this is not always the case. Young people may self-harm without meeting the diagnostic criteria for any mental health condition.

**It would be helpful to explore more what elements of existing evidence-based interventions and approaches have informed the current intervention. What is the framework other than co-production? The co-production process could be described to show how this led to the development of COPING.**

 We thank the reviewer for this point. We have clarified that COPING was developed using a combination complex intervention development approach which brought together both evidence and theory and partnership approaches in the introduction.

 We are preparing manuscripts about COPING’s development and the co-production activity that occurred for submission to journals so we will not describe this in detail here.

 We do though under the ‘intervention’ section of the methods describe in detail the framework and methods that were used to develop COPING.   

**Methods: Eligibility criteria**

**How will the authors define self-harming?**

 We already state in the first sentence under eligibility criteria subheading that self-harm is defined according to the definition of NICE. 

**I wonder what is the role of carers in the process e.g., parents and how they can be involved at least in the qualitative phase of the study.**

 The design of this study was informed by carers of young people who have self-harmed. We have added this to the section about PPI in the manuscript to be explicit. We also state in this section that carers supported the development of COPING, and that they also are supporting the delivery of the feasibility study.  

**A table outlining the design and format of the intervention would be helpful and also the 8 behaviour change techniques and how these will be applied across the two consultations.**

 While we agree that this may be helpful, we wish not to include this level of detail in this manuscript because these are intended to be described in the planned COPING-ID manuscripts which will be submitted shortly. In the second paragraph under ‘Intervention’ in the Methods we describe in detail the 8 behaviour change techniques and what happens in both appointments.

**Patient and Public Involvement (PPI)**

**This section could include further information about the Steering Groups and how the content of the intervention was developed.**

 We have now added more information about the PPI groups in terms of numbers and composition and also how the study specific PPI group supported the development of COPING in the PPI section of the manuscript.

# Reviewer report: Reviewer response for version 1

**DOI:** 10.3310/nihropenres.14737.r31763

**Published:** 2024-08-23

**Authors:** Karen Rodham

**Affiliations:** 1 Department of Health Psychology, University of Chichester, Chichester, England, UK

The researchers present a strong argument for a study to test the feasibility of an intervention that has been designed in collaboration with service users and GPs.

 The suicide prevention in England strategy highlights the need for tailored support to young people who have self-harmed: a high-risk priority group. This proposed intervention developed by the research team could make an important contribution to this clear identified National need.

 This protocol was clearly written. My job as a reviewer was made easy because of the care and attention that had been put into the protocol structure and construction. There was a strong narrative arc throughout and excellent use was made of signposting to keep the reader on track.

*The only element of the document that could be improved in my opinion,* is the plain English summary. There is much scope to simplify the language used here. 

**Rationale: **The rationale for the intervention and for GPs to deliver the intervention was clearly and succinctly shared. The intervention itself has been co-created with collaborative input from both GPs and service users. It now needs to be ‘road-tested’ to learn about the feasibility of embedding the intervention in GP practices.

**Objectives:** The objectives were clear and appropriate.

**Methods:  **The methods were clearly explained, transparent and replicable. The team have employed recognised behaviour change models to inform the design of the intervention. Clear target behaviours have been detailed (the young person using new skills and the GP using the intervention in usual NHS practice). Data collection approaches and analysis measures are explained and a rationale offered for their inclusion.

**PPI **– has clearly been embedded in this proposal from the design of the original intervention, through to the proposed design of the feasibility study. Continued PPI is built into the proposal.

**Risks** have been considered and two risk protocols developed to ensure participants’ safety and wellbeing.

**Bigger picture **– through PPI involvement and team expertise and experience, they have carefully considered the practicalities of maximising the possibility that this intervention will be deliverable in usual NHS practice. 

***In my opinion, this is an important proposal. It has been extremely well-presented and makes a strong case for the work to be supported.***

Is the study design appropriate for the research question?

Yes

Is the rationale for, and objectives of, the study clearly described?

Yes

Are sufficient details of the methods provided to allow replication by others?

Yes

Are the datasets clearly presented in a useable and accessible format?

Not applicable

Reviewer Expertise:

I have experience of researching young people who self-harm. I am a qualitative researcher with a track record of exploring how we can better help people to live well in spite of complex (multiple) physical health conditions.

I confirm that I have read this submission and believe that I have an appropriate level of expertise to confirm that it is of an acceptable scientific standard.

# Response

**Published:** 2024-10-10

**Authors:** Faraz Mughal

**Affiliations:** School of Medicine, Keele University, Keele, England, UK

We would like to thank the reviewer for their positive comments on our submission. In response to their suggestion, we have reworded the plain English summary to make the language used simpler, and so hopefully more readable for the public.

 The plain English summary was not in the downloaded manuscript document so I have pasted it into the new submitted version after the scientific abstract.
